# Updating Predictive Model for Cisplatin-Induced Acute Kidney Injury: Incorporating Concomitant Medications and the Standard Supportive Care

**DOI:** 10.1155/ijne/6615898

**Published:** 2025-10-14

**Authors:** Kazuki Saito, Satoru Nihei, Junichi Asaka, Kenzo Kudo

**Affiliations:** ^1^Department of Pharmacy, Iwate Medical University Hospital, 2-1-1 Idaidori, Yahaba-Cho, Shiwa-Gun, Iwate 028-3695, Japan; ^2^Division of Clinical Pharmaceutics and Pharmacy Practice, Department of Clinical Pharmacy, School of Pharmacy, Iwate Medical University, 1-1-1 Idaidori, Yahaba-Cho, Shiwa-Gun, Iwate 028-3609, Japan

## Abstract

**Background and Objective:**

Cisplatin-induced acute kidney injury (C-AKI) is detrimental to adequate cancer treatment. While scoring systems to predict C-AKI are available, they do not account for the impact of concomitant medications. This study aimed to enhance the predictive model by incorporating concomitant medications as a new predictor.

**Methods:**

We included data from 1785 patients who received cisplatin at Iwate Medical University Hospital between April 2014 and March 2023. Initially, we assessed the performance of the existing model in our cohort. We then explored additional predictors to improve their discriminatory ability guided by the Akaike information criterion. Candidates for new predictors were concomitant anticancer and supportive care medications that were previously unexamined. Finally, we assessed the statistical usefulness of the updated model using the C-statistic and its clinical usefulness using net reclassification improvement (NRI) and decision curve analysis (DCA).

**Results:**

The discriminatory power of the existing model was poor, with a C-statistics of 0.621 (95% confidence interval [CI]: 0.582–0.660). Incorporating magnesium supplementation as a novel predictor significantly improved the model's performance, increasing the C-statistic to 0.695 (95% CI: 0.660–0.731). The updated model demonstrated a superior NRI of 0.143 (95% CI: 0.043–0.243). In the DCA, the updated model yielded higher net benefits for most threshold probabilities.

**Conclusion:**

The existing model did not demonstrate satisfactory clinical performance in our cohort. While incorporating magnesium supplementation significantly improved model discrimination, its status as standard care limits its utility as a predictive variable. These findings underscore the necessity of developing C-AKI prediction models within cohorts receiving uniform, contemporary supportive care regimens.

## 1. Introduction

Cisplatin (CDDP) is a critical agent for the treatment of various malignancies and significantly extends overall survival [1]. However, its administration is associated with nephrotoxicity, which results in CDDP-induced acute kidney injury (C-AKI). Risk factors for C-AKI include age [2, 3], dosage (single and cumulative doses) [4], coexisting hypertension [5], cardiovascular disease [4] or diabetes [6], hepatic cirrhosis [7] and hypoalbuminaemia [2]. Established means of reducing C-AKI include hydration, diuresis with mannitol or furosemide and magnesium supplementation [8]. However, even with these established approaches, C-AKI occurs in 20%–30% of patients receiving CDDP [9, 10] and in approximately 10% even at the first exposure [11, 12]. C-AKI is associated with poor long-term renal prognosis [13, 14] and may cause withdrawal from optimal cancer treatment. In addition, AKI during cancer treatment leads to longer hospital stays and increased healthcare costs [15, 16]. Therefore, it is important to quantitatively assess the risk of developing C-AKI prior to CDDP administration.

Motwani et al. developed a predictive model for estimating the C-AKI risk by combining various risk factors [11]. Their model is scored based on four factors: age, CDDP dose, hypoalbuminaemia and hypertension (Table 1), and their simple scoring allows for the quantitative assessment of C-AKI risk. Their model was created using data from cancer patients at a single facility in the USA (*n* = 2118) and demonstrated moderate discriminatory ability (C-statistic: 0.70–0.72). Burns et al. also validated this scoring system in outpatients and reported that it was possible to stratify C-AKI risk [10]. However, Motwani et al. mentioned the lack of consideration of concomitant medications as a limitation of their study. These factors may be important determinants of C-AKI, and the addition of concomitant medications to the model is expected to increase its discriminatory power and generalisability of the existing model. This study aimed to enhance the predictive model by incorporating concomitant medications as a new predictor. First, we validated the performance of the existing model in our population. Subsequently, candidates for new predictors were explored, and variables with high incremental values were added to the model.

## 2. Materials and Methods

The method described in this article follows the Transparent Reporting of a Multivariable Prediction Model for Individual Prognosis or Diagnosis (TRIPOD) statement [17]. The checklist is presented in Table S1.

### 2.1. Study Design and Data Source

Data of patients who received chemotherapy containing CDDP at Iwate Medical University Hospital (Iwate, Japan) from April 2014 to March 2023 were included in the study. All patients received furosemide or mannitol (or both) as diuretics on the day of CDDP administration. From the identified patients receiving CDDP, those receiving split or weekly doses were excluded, and the following exclusion criteria were applied to the remaining population: (1) CDDP administration before the study period or at other hospitals, (2) lack of information on outcomes, (3) age < 20 years, (4) haematologic malignancies, (5) interruption of administration due to anaphylaxis and (6) refusal to participate in the study. Because the event rate was expected to be low, we included all available data to update the model.

### 2.2. Data Selection

The following items were collected retrospectively from the electronic medical record: patient background (age, sex, primary tumour and stage, height, weight, body surface area and body mass index [BMI]), laboratory tests (serum albumin and serum creatinine [Scr]), CDDP dosage, comorbid diabetes and hypertension and concomitant medications (anticancer drugs, magnesium supplementation, continued use of nonsteroidal anti-inflammatory drugs [NSAIDs] and use of renin–angiotensin system [RAS] inhibitors). The volume of infusions administered on the same day as CDDP was also calculated, and administration of less than 3 L and over less than 5 h was defined as short hydration (SH) with reference to the protocol of Hase et al. [18]. The estimated creatinine clearance (CLcr) and estimated renal glomerular filtration rate (eGFR) were calculated using the Cockcroft–Gault formula [19] and the formula developed by the Chronic Kidney Disease Epidemiology Collaborative, respectively [20]. Pre-existing CKD was defined as eGFR less than 60 mL/min/1.73 m^2^ before CDDP administration. No data were missing in the final analysis, as all collected items were routinely available in the electronic medical records.

### 2.3. Outcome

The primary outcome was the occurrence of C-AKI. We defined C-AKI as a ≥ 0.3 mg/dL increase in Scr within 14 days of the first administration based on Common Terminology Criteria for Adverse Events Version 4.0 as in previous studies [11]. Baseline Scr was defined as the value closest to the first CDDP administration date within 30 days before the administration.

### 2.4. Statistical Analysis

All statistical analyses were performed using R ver4.3.1 (The R Foundation for Statistical Computing, Vienna, Austria) or EZR [21] ver.1.55 (Saitama Medical Center, Jichi Medical University, Saitama, Japan). EZR is a graphical user interface for R. More precisely, it is a modified version of the R commander designed to add statistical functions frequently used in biostatistics.

In the univariate analysis, continuous variables are presented as the median and interquartile range (IQR), and categorical variables are presented as the number of applicable samples (percentages). Fisher's exact test was used for the univariate analysis of categorical variables, and the Mann–Whitney *U* test was used for the univariate analysis of continuous variables. A logistic regression model was used for multivariate analysis. While univariate analysis was performed on continuous variables to explore their associations, for the purpose of examining the effects of concomitant medications, new candidate predictors for the multivariate model were exclusively treated as binary variables indicating the presence or absence of concomitant medications. The Motwani risk score was treated as a continuous variable in all models. As our model updating strategy was based on adding new candidate predictors one-by-one to the existing Motwani score to evaluate their incremental value (Table2), the assessment of multicollinearity was primarily focused on the relationship between the existing score and each new predictor. However, even in models that combined multiple predictors (e.g. the existing model with magnesium and another candidate), we confirmed that all VIF values remained below 3, indicating no significant multicollinearity. The significance level was set at *p* < 0.05.

### 2.5. Performance Evaluation of the Existing Model

We evaluated the performance of the existing model in predicting C-AKI in our population. We calculated the probability of C-AKI using the existing model, which assigns a total score based on four factors: age, CDDP dose, hypoalbuminaemia and hypertension (Table 1). To reproduce the original model's reported performance (C-AKI probability of 0.04 at score 0 and an odds ratio [OR] of 1.49 per score point [11]), we derived the logistic regression coefficients and subsequently used a coefficient of 0.399 for the score and −3.178 for the intercept. The model's overall fit was evaluated using *R*^2^ (Cox–Snell and Nagelkerke), Brier score, calibration performance by calibration-in-the-large, calibration slope, observed/expected ratio (O/E ratio), integrated calibration index (ICI) and discriminant performance using the C-statistic [22]. To obtain robust estimates and 95% confidence intervals (CIs) for these performance metrics, each index was evaluated using 10,000 bootstrap samples.

### 2.6. Model Update

In this study, the model was updated by what Nieborer et al. call ‘recalibration and extension' [23]. This method estimates the regression coefficients by fitting the logistic regression model to the overall linear predictors of the original and new models. The original model was considered as one predictor overall, and the regression coefficients for the individual predictors were not re-estimated. In this study, candidates for new predictors were concomitant medications with known nephrotoxicity, such as doxorubicin (DXR), methotrexate (MTX), pemetrexed (PEM), gemcitabine (GEM), immune checkpoint inhibitors (CPIs; nivolumab and pembrolizumab), vascular endothelial growth factor (VGEF) inhibitors (bevacizumab) and those that significantly increased the risk of C-AKI in univariate analysis. Magnesium supplementation and SH, which are considered to be associated with CDDP-induced nephrotoxicity, were also included as candidates. We incorporated these individually into the existing model and examined their incremental values. The incremental value was assessed using the Akaike information criterion (AIC), a statistical measure that balances model fit and complexity. A change in the AIC greater than 10 was considered significant [24]. Once the new predictor was determined, we estimated the regression coefficients of the scores of the existing model and the new predictor using logistic regression. The score of the new predictor was determined by dividing the OR of the new predictor by the OR of the existing score (as per one point) and rounded to the nearest 0.5 increments. The performance of the updated scoring model was evaluated using the same measures as in the existing model. The regression coefficients and model performance were calculated using the bootstrap sample, and the mean and 95% CI were estimated after 10,000 iterations.

### 2.7. Internal Validation of the Updated Model

We used a bootstrap method for internal validation of our scoring model [25]. The model creation process was repeated 10,000 times using a bootstrap sample. The optimism was calculated by comparing the C-statistic in the population with that in the bootstrap sample of the prediction model developed based on the bootstrap sample. The optimism-corrected predictive performance was calculated by subtracting the mean of optimism from the original C-statistic.

### 2.8. Measuring the Usefulness of the Updated Model

We quantitatively assessed the usefulness of the updated model in terms of net reclassification improvement (NRI) and integrated discriminability improvement (IDI): NRI is the number of correct reclassifications per outcome presence, and IDI is the difference in predicted probability between those with and without the outcome. NRI was evaluated by grouping the incidence of C-AKI in the existing model into less than 10% (low risk), 10%–20% (intermediate risk) and 20% or more (high risk).

We also used a decision curve analysis (DCA) to assess the clinical usefulness of the model revisions. This method plots the net benefit on the vertical axis and the threshold probability on the horizontal axis. The net benefit represents the true positive benefit minus the false positive harm, where the latter is weighted by the relative harm of a false positive compared to the benefit of a true positive at a given threshold probability. The threshold probability is defined as the point at which the expected benefit of an intervention equals the expected benefit of avoiding that intervention. Once a threshold is determined, the higher the net benefit, the better the model.

## 3. Result

**Figure 1:**
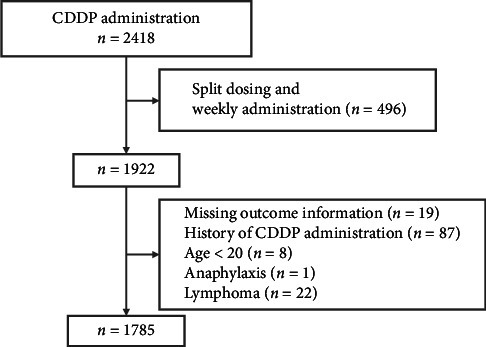
Flowchart of the cohort selection used to update the model.

We identified 2418 patients who underwent planned CDDP administration and excluded 496 who received split or weekly administration. As a result of applying the exclusion criteria in Figure 1 to the remaining 1922 patients, 1785 patients were included in the final analysis (Figure 1). C-AKI occurred in 205 patients (11.5%) and was classified according to the Acute Kidney Injury Network (AKIN) criteria: Stage 1 (less than 2.0-fold increase in Scr from baseline) in 161 patients (9.0%), Stage 2 (2.0- to 3.0-fold increase) in 38 patients (2.1%) and Stage 3 (> 3.0-fold increase) in six patients (0.3%).

Patient characteristics and univariate analyses are shown in Table 3. Patients tended to be older (66 years [IQR: 59–71 years]), predominantly male (*n* = 1231 [69.0%]) and had a normal BMI (21.8 kg/m^2^ [IQR: 19.5–24.3 kg/m^2^]). The primary tumour types observed in our cohort predominantly included head and neck cancer (587 patients, 32.9%), oesophageal cancer (373 patients, 20.9%), lung cancer (222 patients, 12.4%), urothelial carcinoma (183 patients, 10.3%) and gastric cancer (7.6%), reflecting common malignancies treated with CDDP. Baseline renal function was good (eGFR was 76.5 mL/min/1.73 m^2^ [IQR: 70.0–82.9 mL/min/1.73 m^2^]) with some exceptions. The C-AKI group was significantly older (OR: 1.02 [95% CI: 1.01–1.04]), male (OR: 2.06 [95% CI: 1.43–2.96]) and had a higher BMI (OR: 1.07 [95% CI: 1.03–1.11]), comorbid hypertension (OR: 2.01 [95% CI: 1.50–2.70]) and lower baseline eGFR (OR: 0.99 [95% CI: 0.97–1.00]). The C-AKI group was significantly more likely to receive capecitabine (OR: 7.77 [95% CI: 1.09–55.50]), GEM (OR: 3.30 [95% CI: 2.28–4.77]), MTX (OR: 10.50 [95% CI: 2.32–47.10]), S-1 (OR: 1.91 [95% CI: 1.18–3.09]) and etoposide (OR: 1.98 [95% CI: 1.01–3.09]). Patients who were not administered magnesium had a significantly higher probability of developing C-AKI (OR: 3.75 [95% CI: 2.71–5.15]).

We tested the performance of the existing model for our population (Table 4): The Cox–Snell *R*^2^ was −0.010 (95% CI: −0.061–0.037), Brier score was 0.100 (95% CI: 0.089–0.110), O/E ratio was 0.729 (95% CI: 0.641–0.820), calibration slope was 0.603 (95% CI: 0.420–0.793) and ICI was 0.047 (95% CI: 0.035–0.060). The C-statistic, a measure of discriminability, was 0.621 (95% CI: 0.582–0.660).

We then added a new candidate predictor to the existing model, as shown in Table 2. Candidate variables included concomitant anticancer agents with known nephrotoxicity (DXR, MTX, PEM, GEM, CPIs and VGEF inhibitors) and those that were significant in the univariate analysis (capecitabine, S-1 and etoposide). Supportive therapies expected to be related to the likelihood of developing C-AKI (magnesium supplementation or SH) were added to the model. Among the candidate variables, ‘no magnesium supplementation' showed the smallest AIC and the largest improvement in AUC (0.710). Notably, adding GEM alone to the existing model also yielded a significant improvement in AIC and discrimination. However, incorporating GEM in addition to magnesium did not provide significant further improvement (Table 2). We considered adding further predictors, but no variable significantly altered the AIC. Therefore, ‘no magnesium supplementation' was selected as the single most impactful additional predictor.

**Figure 2:**
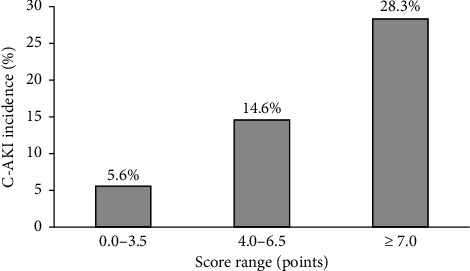
Incidence of cisplatin-induced acute kidney injury (C-AKI) across score ranges.

**Figure 3:**
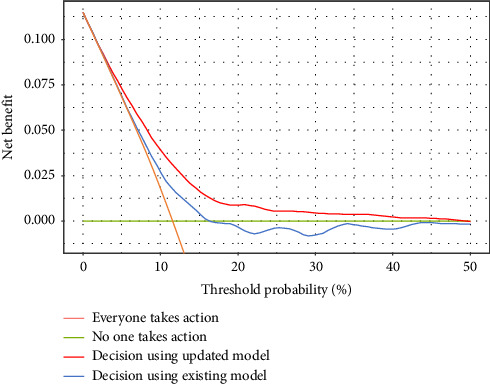
Decision curve analysis. The orange line shows the net benefit if everyone takes action to avoid C-AKI, and the green line shows the net benefit if no one takes action. The red and blue lines show the net benefit of using the updated and existing models, respectively, to decide whether to take action to avoid C-AKI. A DCA illustrating the full range of probability thresholds (0%–100%) is provided in Supporting Figure S2.

The OR for ‘no magnesium supplementation' was 3.91 (95% CI: 2.83–5.26), the OR per one point of the existing score was 1.29 (95% CI: 1.19–1.40), and the intercept OR was 0.04 (95% CI: 0.02–0.05). We assigned a score of ‘no magnesium supplementation' as 3.0, and the risk score for the updated model was calculated by adding the score for ‘no magnesium supplementation' to the risk score of the existing model (Table 1). The OR for each one-point increase in the updated score was 1.38 (95% CI: 1.29–1.48). C-AKI incidence rates were 5.6%, 14.6% and 28.3% when the updated model scored 0–3.5, 4–6.5 and 7 points, respectively (Figure 2). The updated model showed a better calibration and fit than the existing model (Table 4 and Figure S1). The C-statistic of the updated scoring model was 0.695, which was higher than that in a previous study (Table 4). The optimism estimated by bootstrapping was 0.0012, with a corrected C-statistic of 0.694 (95% CI: 0.657–0.730). The updated scoring model improved the reclassification to 0.143 (95% CI: 0.043–0.243) for NRI and 0.019 (95% CI: 0.008–0.029) for IDI (Table 5). The DCA of the existing and updated scoring models is shown in Figure 3. For most threshold probabilities below 50%, the updated model produces higher net benefits than the existing scoring model.

## 4. Discussion

In our study, C-AKI occurred in 11.5% of patients at the time of initial CDDP administration, suggesting that C-AKI is a common problem in patients receiving CDDP. AKI during chemotherapy can lead to prolonged hospital stays, increased healthcare costs, reduced quality of life, shortened survival and increased risk of future CKD [13–16, 26]. Additionally, AKI can be missed during chemotherapy, leading to delays in addressing it [27]. To avoid or detect C-AKI early, it is desirable to establish a tool to quantitatively assess the risk of C-AKI prior to CDDP administration.

The existing scoring model for predicting the risk of C-AKI, proposed by Motwani et al., is considered simple and clinically useful. However, our results showed that the overall model fit and calibration decreased in our cohort. The calibration slope and O/E ratios indicated that the existing model overestimated C-AKI risk in our population (Table 4). Reduced model fit and calibrability are not surprising and can be improved by recalibrating the model. Our population was characterised by older age and lower BMI than those in the existing model (Table 3). These differences, as well as the racial differences in susceptibility to C-AKI, may have contributed to the decline in the model fit. However, it should be noted that the discriminative power was significantly reduced. This suggests the presence of predictors not included in the existing model. Based on these results, we decided to include new predictors in the model update.

A major limitation of the existing model is that it does not consider the effects of concomitant drugs. CDDP is rarely administered alone but in combination with several anticancer drugs. Some of these anticancer drugs are nephrotoxic on their own, and their effects need to be clarified. The presence or absence of drugs administered for renal protection may also be an important factor influencing the development of C-AKI. We hypothesised that adding these concomitant drugs (anticancer and supportive care drugs) to the model would improve the predictive performance of the existing model for C-AKI and searched for explanatory variables that would contribute to an improved predictive model.

The results showed that magnesium supplementation was a major determinant of C-AKI and that adding it to the model improved its discriminative ability. The updated model showed a good fit and calibration performance (Table 4 and Figure S1). It also showed a significant improvement in the discriminative ability compared with the existing model. The NRI results showed that the updated model significantly improved the reclassification of the group in which C-AKI did not occur. This is also related to the fact that the existing model overestimates the risk of C-AKI. This indicates that the updated model adequately predicted the risk of C-AKI compared to the existing model, thereby offering improved potential for clinical application.

Building upon this improved predictive ability, while the scoring system itself provides quantitative risk assessment, its primary value lies in potentially informing clinical decision-making to mitigate C-AKI. Previous studies highlight that even a mild increase in Scr, indicative of C-AKI, can negatively impact mortality and long-term renal function [28, 29]. Based on these risk strata, it is conceivable that for patients categorised as high-risk (≥ 7 points), alternative platinum agents (e.g. carboplatin or oxaliplatin) or CDDP dose adjustments, along with intensified monitoring, could be considered. For those at intermediate risk (4.0–6.5 points), strict adherence to nephroprotective measures, including optimised hydration, may prove prudent. Furthermore, if magnesium supplementation is not yet planned for any patient, its addition should be strongly considered regardless of the risk category. However, the direct application of these specific risk thresholds for immediate clinical intervention requires further prospective validation.

Contrary to our expectations, the concomitant use of nephrotoxic anticancer agents was not a predictor of C-AKI. The principle of combining anticancer drugs is to minimise toxicity and maximise antitumour effects; thus, regimens are typically designed to avoid overlapping toxicities. For instance, in a study of CDDP plus PEM, a common combination, no difference in the incidence of renal toxicity was observed between the PEM and placebo groups [30]. Beyond this general principle, several factors may explain why some concomitant agents, despite showing univariate associations (e.g. GEM and MTX in Table 3), did not emerge as independent predictors in the multivariate model. First, the apparent univariate association of these agents with C-AKI might have been due to confounding by the magnesium supplementation status. In our additional analysis, there was a negative correlation between the proportion receiving magnesium supplementation and the OR for C-AKI (Figure S3). Second, for agents such as MTX (*n* = 7) and capecitabine (*n* = 4) with very low prevalence, their limited number of exposed events likely restricted their ability to significantly improve the overall model's discriminatory power, even if individual effects were present. Thus, in populations with a higher prevalence of specific concomitant nephrotoxic agents, these agents might contribute more substantially to predictive models. It is also possible that there may be effects of complications or concomitant use that we were unable to measure. Larger prospective studies with more diverse cohorts are warranted to fully clarify the independent predictive contribution of various concomitant anticancer agents to C-AKI risk.

The benefit of the updated model for clinical decision-making was assessed using DCA (Figure 3). The figure plots the net benefit on the vertical axis and the threshold probability on the horizontal axis. The threshold probability is defined as ‘where the expected benefit of treatment is equal to the expected benefit of avoiding treatment'. Here, it can be interpreted as the probability of C-AKI occurring when the benefit of taking action to avoid C-AKI (e.g. avoiding CDDP administration) is equal to the harm of administering CDDP without taking action to avoid C-AKI. Simply put, the farther to the left on the horizontal axis, the more important the avoidance of C-AKI is than cancer treatment. On this axis, we plotted the net benefit curve for the condition ‘everyone takes (or does not take) action to avoid C-AKI' and the net benefit curve when the score model was used. The results showed that the updated model yielded the highest net benefit across threshold probabilities ranging from approximately 2.5% to less than 50%. Because cancer treatment is usually given priority, it is assumed that some risk of C-AKI is acceptable and that the threshold probability is greater than 2.5%. Therefore, decision-making using the updated model makes the most sense in the general clinical context.

However, adding this predictor may not be entirely successful because magnesium supplementation is now a standard supportive therapy that should be provided to all patients. Evidence consistently shows that magnesium supplementation reduces the nephrotoxicity of CDDP, reinforcing its importance [31–34]. Therefore, in the current healthcare context, including ‘no magnesium supplementation' as a predictor has limited significance. The reason why not all members of our cohort received magnesium supplementation was that it included patients who were treated before magnesium supplementation became widely available. Other predictors should be explored to meaningfully enhance the model. Addressing this issue in future studies could involve developing models for populations with uniform supportive care practices.

This study has several limitations. One is the lack of external validation. Due to the relatively low event rate (11.5%), we utilised the entire cohort for model updating to maximise statistical power. While internal validation via bootstrapping was performed, this approach inherently lacks true external validation. External validation in an independent cohort, potentially through multicentre collaboration or temporal validation within our institution using subsequent data, is now critically required to confirm the generalisability of the updated model before clinical implementation. Additionally, while the overall cohort size was substantial, the exploration of predictors with very low prevalence (e.g. MTX *n* = 7, capecitabine *n* = 4) was limited by the small number of exposed events. Findings for these specific agents should be interpreted with caution due to potential instability of estimates [35]. The second concern is group representativeness. This was a single-centre study, and the population used may not necessarily be representative of the population receiving CDDP. This limitation, along with the first, should be addressed by confirming the suitability of the model for other populations.

## 5. Conclusions

Our results revealed that existing C-AKI prediction models lack sufficient discriminatory power in the Japanese population. Updating the model by adding magnesium supplementation as a predictor improved its discriminatory ability. However, since magnesium supplementation is now a standard supportive treatment, its inclusion has limited impact on current clinical practice. These findings suggest that developing a C-AKI prediction model relevant to contemporary clinical practice may require cohorts treated with standardised supportive care.

## Figures and Tables

**Table 1:** The existing model to predict cisplatin-induced acute kidney injury.

Variable	Value	Score
Age	≤ 60 years	0.0
	61–70 years	1.5
	> 70 years	2.5

Albumin	> 3.5 g/dL	0.0
	≤ 3.5 g/dL	2.0

CDDP dose	≤ 100 mg	0.0
	101–150 mg	1.0
	> 150 mg	3.0

Hypertension	No	0.0
	Yes	2.0

**Table 2:** Incremental value of predictors added to the existing models.

Candidate predictor added to the existing model	AUC (existing model + candidate)	AIC (existing model + candidate)	AUC (existing model + Mg + candidate)	AIC (existing model + Mg + candidate)
None	0.622	1240.5	0.71	1175.3
Capecitabine	0.625	1239.7	0.71	1176.1
DXR	0.625	1240.9	0.708	1176.6
Etoposide	0.629	1239.4	0.718	1170.4
GEM	0.666	1208.6^∗^	0.710	1176.6
MTX	0.633	1232.2	0.712	1172.6
PEM	0.624	1241.0	0.710	1177.3
S-1	0.636	1235.1	0.709	1177.3
CPIs	0.628	1236.5	0.713	1173.1
VEGF	0.622	1242.5	0.709	1176.8
Mg	0.71	1175.3^∗^	—	—
SH	0.624	1239.2	0.711	1177.3

*Note:* Changes in the C-statistics and the Akaike information criterion (AIC) when variables are added to the existing model are shown. The left column (variable) lists the names of added predictors. Significant changes in the AIC are marked with asterisks. Because the existing model with Mg had the lowest AIC, we considered adding further predictors (right side). The model ‘Existing Model + Mg' (AUC 0.71, AIC 1175.3) is listed under the ‘None' row for the right section. None: Value when no variables were added; DXR: doxorubicin, GEM: gemcitabine, MTX: methotrexate, PEM: pemetrexed, VGEF: vascular endothelial growth factor inhibitor, CPIs: immune checkpoint inhibitors, Mg: magnesium supplementation.

Abbreviation: SH, short hydration.

**Table 3:** Univariate analysis.

	Overall (*n* = 1785)	No C-AKI (*n* = 1580)	C-AKI (*n* = 205)	Odds ratio (95% CI)	*p* value
Age, years	66 (59–71)	66 (58–71)	68 (61–73)	1.02 (1.01–1.04)	0.001
Male sex	1231 (69.0)	1065 (67.4)	166 (81.0)	2.06 (1.43–2.96)	< 0.001
Body weight (kg)	57.6 (50.1–65.5)	57.0 (50.0–65.0)	60.8 (53.5–68.6)	1.02 (1.01–1.04)	< 0.001
BMI (kg/m^2^)	21.8 (19.5–24.3)	21.7 (19.4–24.2)	22.4 (20.4–25.4)	1.07 (1.03–1.11)	0.001
DM, *n* (%)	234 (13.1)	201 (12.7)	33 (16.1)	1.32 (0.88–1.97)	0.187
HT, *n* (%)	663 (37.1)	556 (35.2)	107 (52.2)	2.01 (1.50–2.70)	< 0.001
Recurrence/metastasis	607 (34.0)	535 (33.9)	72 (35.1)	1.06 (0.78–1.43)	0.754
CDDP dosage (mg)	110 (90–128)	110 (90–128)	110 (93–129)	1.01 (1.00–1.01)	0.119
ALB (mg/dL)	3.9 (3.5–4.2)	3.9 (3.5–4.2)	3.8 (3.3–4.2)	0.78 (0.60–1.01)	0.123
Creatinine (mg/dL)	0.7 (0.6–0.9)	0.7 (0.6–0.9)	0.8 (0.7–0.9)	2.83 (1.32–6.06)	0.026
CLcr (mL/min)	77.1 (63.4–94.6)	77.4 (63.5–94.6)	75.8 (63.0–94.3)	1.00 (1.00–1.01)	0.803
eGFR (mL/min/1.73 m^2^)	76.5 (70.0–82.9)	76.5 (70.1–83.1)	75.6 (69.4–94.3)	0.99 (0.97–1.00)	0.021
Pre-existing CKD	169 (9.5)	143 (9.1)	26 (12.7)	1.46 (0.94–2.28)	0.099
Prediction score, points	3.5 (2.0–4.5)	3.0 (2.0–4.5)	4.5 (2.5–5.5)	1.27 (1.17–1.38)	< 0.001
Capecitabine	4 (0.2)	2 (0.1)	2 (1.0)	7.77 (1.09–55.5)	0.041
CPA	8 (0.4)	8 (0.5)	0 (0.0)	—	0.608
CPT-11	72 (4.0)	69 (4.4)	3 (1.5)	0.33 (0.10–1.04)	0.056
DTX	600 (33.6)	541 (34.2)	59 (28.8)	0.78 (0.56–1.07)	0.135
DXR	121 (6.8)	107 (6.8)	14 (6.8)	1.01 (0.58–1.80)	1.000
GEM	182 (10.2)	134 (8.5)	48 (23.4)	3.30 (2.28–4.77)	< 0.001
MTX	7 (0.4)	3 (0.2)	4 (2.0)	10.5 (2.32–47.1)	0.004
PEM	100 (5.6)	92 (5.8)	8 (3.9)	0.66 (0.31–1.37)	0.332
PTX	71 (4.0)	71 (4.5)	0 (0.0)	—	0.974
S-1	121 (6.8)	98 (6.2)	23 (11.2)	1.91 (1.18–3.09)	0.011
VNR	65 (3.6)	58 (3.7)	7 (3.4)	0.93 (0.42–2.06)	1.000
VP-16	55 (3.1)	44 (2.8)	11 (5.4)	1.98 (1.01–3.09)	0.048
5-FU	708 (39.7)	643 (40.7)	65 (31.7)	0.68 (0.50–0.92)	0.015
EGFR inhibitor	5 (0.3)	5 (0.3)	0 (0.0)	—	1.000
VEGF inhibitor	56 (3.1)	51 (3.2)	5 (2.4)	0.75 (0.30–1.90)	0.673
CPIs	22 (1.2)	22 (1.4)	0 (0.0)	—	0.166
Short hydration	372 (20.8)	343 (21.7)	29 (14.1)	0.59 (0.39–0.90)	0.0133
No Mg supplement	352 (19.7)	264 (16.7)	88 (42.9)	3.75 (2.71–5.15)	< 0.001

*Note:* Continuous variables are shown as median (IQR) and categorical variables as a number of persons (percentage). HT: hypertension, CDDP: cisplatin, ALB: serum albumin, CLcr: creatinine clearance, CPA: cyclophosphamide, CPT-11: irinotecan, DTX: docetaxel, DXR: doxorubicin, GEM: gemcitabine, MTX: methotrexate, PEM: pemetrexed, PTX: paclitaxel, VNR: vinorelbine, VP-16: etoposide, 5-FU: 5-fluorouracil, CPIs: immune checkpoint inhibitors, Mg: magnesium, VGEF: vascular endothelial growth factor.

Abbreviations: BMI, body mass index; CKD, chronic kidney disease; DM, diabetes mellitus; eGFR, estimated glomerular filtration rate; EGFR, endothelial growth factor receptor.

**Table 4:** Comparison of model performance.

	Existing model Mean (95% CI)	Updated model Mean (95% CI)
Overall fit		
*R*^2^ Nagelkerke	0.040 (0.019–0.068)	0.102 (0.067–0.142)
*R*^2^ Cox–Snell	−0.010 (−0.061–0.037)	0.100 (0.063–0.138)
Brier score	0.100 (0.089–0.110)	0.096 (0.085–0.106)
Calibration		
Calibration-in-the-large	0.111 (0.095–0.126)	−0.004 (−0.148–0.140)
Observed/expected ratio	0.729 (0.641–0.820)	1.001 (0.878–1.122)
Calibration slope	0.603 (0.420–0.793)	1.003 (0.808–1.210)
Integrated calibration index	0.047 (0.035–0.060)	0.016 (0.008–0.025)
Discrimination		
C-statistics	0.621 (0.582–0.660)	0.695 (0.660–0.731)

*Note:* Calibration curves for both models are presented in Supporting Figure S1.

**Table 5:** Performance comparison of the models.

C-AKI group	Updated model		
Existing model	Low risk	Intermediate risk	High risk
Low risk	30	26^a^	0^a^
Intermediate risk	27^b^	43	20^a^
High risk	0^b^	28^b^	31

**No C-AKI group**	**Updated model**		
**Existing model**	**Low risk**	**Intermediate risk**	**High risk**

Low risk	623	74^d^	0^d^
Intermediate risk	243^c^	322	63^d^
High risk	0^c^	189^c^	66

		**C-AKI group**	**No C-AKI group**

Correct reclassification		46^a^	432^c^
Incorrect reclassification		55^b^	137^d^
Net reclassification		−9^a-b^	295^c-d^
NRI		0.143 (0.043–0.243)	
IDI		0.019 (0.008–0.029)	

*Note:* Risk classification using the existing model (Motwani) and the updated model for the C-AKI and no C-AKI groups. C-AKI risk was classified as low risk (< 10%), intermediate risk (10%–20%) and high risk (≥ 20%). In the C-AKI group, correct reclassification is combined^a^ and incorrect reclassification is combined^b^. Similarly, correct reclassification is combined^c^ and incorrect reclassification is combined^d^ in the no C-AKI group.

Abbreviations: IDI, integrated discrimination index; NRI, net reclassification index.

## Data Availability

The data that support the findings of this study are available on request from the corresponding author. The data are not publicly available due to privacy or ethical restrictions. The code of R of this study is available from the corresponding author upon request.

## Supporting Information

This section contains the TRIPOD statement checklist and the existing and updated model calibration curve.
